# Product formulas for basic hypergeometric series by evaluations of Askey–Wilson polynomials

**DOI:** 10.1007/s11139-026-01400-x

**Published:** 2026-08-18

**Authors:** Howard S. Cohl, Michael J. Schlosser

**Affiliations:** 1 Applied and Computational Mathematics Division, National Institute of Standards and Technology, Gaithersburg, MD 20899-8910 USA; 2 Fakultät für Mathematik, Universität Wien, Oskar-Morgenstern-Platz 1, A-1090 Vienna, Austria

**Keywords:** Nonterminating basic hypergeometric series, Transformations, Products of basic hypergeometric series, 33D15, 33D50

## Abstract

Ismail and Wilson derived a generating function for Askey–Wilson polynomials which is given by a product of *q*-Gauss (Heine) nonterminating basic hypergeometric functions. We provide a generalization of that generating function which contains an extra parameter. A special case gives a closed form summation formula for a quadruple basic hypergeometric sum. We further present new terminating balanced ${\phantom {a}}_4\phi _3$ summations that give rise to *q*-quadratic special values for Askey–Wilson polynomials. We also similarly present new terminating 2-balanced and 3-balanced ${\phantom {a}}_4\phi _3$ summations. Using the Ismail–Wilson generating function combined with explicit summations for terminating balanced basic hypergeometric ${\phantom {a}}_4\phi _3$ series, we compute new basic hypergeometric product transformations for nonterminating basic hypergeometric series and provide corresponding integral representations. Further new identities are obtained by applying Cayley–Orr type expansion formulas.

## Introduction

The Askey–Wilson polynomials stand on the top of the *q*-Askey-scheme [21] as they are the most general family of orthogonal polynomials in one variable that can be written as basic hypergeometric series. They are well-studied objects and play a central role in *q*-series and the theory of (*q*-)special functions. The monograph [15] discusses many of the relevant properties of the Askey–Wilson polynomials and related objects, together with detailed proofs. In [17, (1.9)], Ismail and Wilson obtained a generating function for the Askey–Wilson polynomials, see (5) below; this can be used to uniquely determine the coefficients in the three-term relation satisfied by the Askey–Wilson polynomials (see [15, pp. 385–386]).

This paper is devoted to a thorough study of the generating function (5), viewed as an expansion of a product of two nonterminating basic hypergeometric ${\phantom {a}}_2\phi _1$ series in terms of a single sum of the Askey–Wilson polynomials with coefficients being basic hypergeometric terms. One of our main results is a generalization of the Ismail–Wilson generating function in the form of a product formula for two nonterminating basic hypergeometric ${\phantom {a}}_3\phi _2$ series. The expansion here takes the form of a three-fold sum, see Theorem 2.3. An immediate consequence of this formula is an elegant closed form evaluation for a specific quadruple basic hypergeometric sum, given in Corollary 2.4, which we believe is new. In addition, a great portion of our study is devoted to finding applications of the generating function (5) in the context of product formulas for basic hypergeometric series (which we deduce by applying various available summations for terminating balanced ${\phantom {a}}_4\phi _3$ series) and corresponding integral representations.

We believe that our study, which is focused on formulas connecting (products of) ${\phantom {a}}_2\phi _1$ series and balanced terminating ${\phantom {a}}_4\phi _3$ series, is a valuable contribution to the theory of basic hypergeometric series. These functions are two of the most important families of *q*-special functions. They satisfy important properties including difference equations that extend the hypergeometric differential equation, and in particular, their appearance in the theory of basic hypergeometric orthogonal polynomials, cf. [15]. We hope that the identities given here will be useful, for instance in studies related to combinatorial representation theory, where product and linearization formulas typically appear.

Before turning to the Askey–Wilson polynomials in Sect. 2, we recall some basic notions from the theory of basic hypergeometric series in the following Sect. 1.1. Further material concerns an overview of ${\phantom {a}}_4\phi _3$ summations in Sect. 3, and, as applications, the nonterminating product transformations in Sect. 4 and a short conclusion is given in the end in Sect. 5.

### Preliminaries

We adopt the following set notations: $\mathbb {N}_0:=\{0\}\cup \mathbb {N}=\{0, 1, 2,...\}$, and we use the sets $\mathbb {Z}$, $\mathbb {R}$, ${{\mathbb {C}}}$ which represent the integers, real numbers and complex numbers respectively, and further write ${{\mathbb {C}}^*}:={{\mathbb {C}}}\setminus \{0\}$. Throughout the paper, we assume that the empty sum vanishes and the empty product is unity. We also adopt the following conventions for succinctly writing elements of lists. To indicate sequential positive *and* negative elements and powers, we write

$$ \{\!\pm \!\}a:=\{a,-a\} \qquad \text {and}\qquad z^{\{\!\pm \!\}}:=\{ z,z^{-1}\}. $$

In order to obtain our derived identities, we rely on properties of the *q*-Pochhammer symbol (a.k.a. the *q*-shifted factorial). For any $n\in \mathbb {N}_0$, $q,a \in {{\mathbb {C}}}$, the *q*-Pochhammer symbol is defined as

$$ (a;q)_n:=(1-a)(1-aq)\cdots (1-aq^{n-1}), \qquad \text {and }\qquad (a;q)_\infty :=\lim _{n\rightarrow \infty }(a;q)_n, $$

where in the infinite product we require |q|<1 for absolute convergence. We will also use, for $k\in \mathbb {N}$ and $n\in \mathbb {N}_0\cup \{\infty \}$, the common notational product convention

$$ (a_1,...,a_k;q)_n:=(a_1;q)_n\cdots (a_k;q)_n. $$

The *q*-Pochhammer symbol satisfies a number of elementary identities (cf. [13, Appendix I]) such as

1 $$\begin{aligned} (a^2;q^2)_n=(\{\!\pm \!\}a;q)_n, \end{aligned}$$

which we tacitly use throughout this paper. Some other useful identities for the *q*-shifted factorial for $n\in \mathbb {N}_0$, are

$$\begin{aligned}  &   (a;q)_{n+1}=(1-a)(qa;q)_n,\\  &   (a;q)_{n-1}=\frac{(q^{-1}a;q)_n}{(1-q^{-1}a)},\\  &   \frac{(1-q^na)}{(1-a)}=\frac{(qa;q)_n}{(a;q)_n},\\  &   (a;q^{\frac{1}{2}})_n=\left\{ \begin{array}{ll} \displaystyle (a,q^\frac{1}{2}a;q)_{\frac{n}{2}} &  \qquad \textrm{if}\ n\ \textrm{even},\\ \displaystyle (1-a)(q^{\frac{1}{2}}a,qa;q)_{\frac{n-1}{2}} &  \qquad \textrm{if}\ n\ \textrm{odd}, \end{array} \right. \\  &   (a;q)_{2n}=(a,qa;q^2)_n=(\{\!\pm \!\}\sqrt{a},\{\!\pm \!\}\sqrt{qa};q)_n. \end{aligned}$$

The basic hypergeometric series, which we will often use, is defined for $q,z\in {{\mathbb {C}}^*}$, such that |q|<1, $s,r\in \mathbb {N}_0$, $b_j\not \in q^{-\mathbb {N}_0}$, j=1,...,s, as [21, (1.10.1)]where for r=s+1 we assume |z|<1 for convergence, if the series does not terminate. (For r≤s the series converges for any *z*, due to the quadratic powers of *q* appearing in the terms of the series.) We refer to a basic hypergeometric series as ℓ*-balanced* if $q^\ell a_1\cdots a_r=b_1\cdots b_s$, and *balanced* (or Saalschützian) if ℓ=1.

The basic hypergeometric function ${\phantom {a}}_r\phi _s$ is a *q*-extension of the generalized hypergeometric function ${\phantom {a}}_rF_s$, defined for $z,a_1,\ldots ,a_r\in \mathbb {C}$ and $b_1,\ldots ,b_s\in \mathbb {C}\setminus \{0,-1,-2,\ldots \}$ as [25, (16.2.1)]where $(a)_k=a(a+1)\cdots (a+k-1)$ is the shifted factorial. For the conditions of absolute or conditional convergence of the ${\phantom {a}}_rF_s$ series, in case it does not terminate, see [25, Sect. 16.2].

## The Askey–Wilson polynomials

The Askey–Wilson polynomials [15, Chap. 15] form the most general family of orthogonal polynomials in *x* that are also basic hypergeometric series. They contain, in addition to the variable *x* and the base *q*, four extra parameters *a*, *b*, *c*, *d*. The *n*-th degree Askey–Wilson polynomial is denoted by $p_n(x;a,b,c,d|q)$. These polynomials are symmetric in the four variables *a*, *b*, *c*, *d* and have the following terminating balanced ${\phantom {a}}_4\phi _3$ basic hypergeometric series representations [10, (15)] (with obvious additional ones corresponding to the symmetries in the four variables), see [10, Theorem 7].

### Theorem 2.1

Let $n\in \mathbb {N}_0$, $x=\frac{1}{2}(w+w^{-1})$, and $q,w,a,b,c,d\in {{\mathbb {C}}^*}$. Then234

An important product generating function for Askey–Wilson polynomials was derived by Ismail–Wilson, namely [17, (1.9)]5where $x=\frac{1}{2}(w+w^{-1})$. Comparison of the coefficients of $t^n/(q,ab,cd;q)_n$ on both sides of (5) reveals that the Askey–Wilson polynomials satisfy the convolution formula

6 $$\begin{aligned} p_n(x;a,b,c,d|q)=(q,ab,cd;q)_n\sum _{j=0}^n\frac{(aw,bw;q)_j}{(q,ab;q)_j} \frac{(\frac{c}{w},\frac{d}{w};q)_{n-j}}{(q,cd;q)_{n-j}}w^{n-2j}, \end{aligned}$$

which, after some elementary manipulations of *q*-Pochhammer symbols is just (4). (This shows that (6) and (4) are equivalent.)

### Remark 2.2

A referee pointed out that (6) with $w={\mathrm e}^{i\theta }$ yields for a=b=c=d=0 the well-known formula for continuous *q*-Hermite polynomials given at the end of [21, Section 14.26]. This is interesting because, for instance in the standard *q*-hypergeometric expression for the Askey–Wilson polynomials (2), one cannot specialize a=b=c=d=0.

### A triple sum generating function for Askey–Wilson polynomials

We have the following triple summation generating function for Askey–Wilson polynomials which generalizes the Ismail–Wilson product generating function (5) by an extra parameter *u*.

#### Theorem 2.3

Let |q|<1 and $u,w,t,a,b,c,d\in {{\mathbb {C}}^*}$ such that |u|<min(|a|,|c|), $|t|<\min (|w|,|w^{-1}|)$, $x=\frac{1}{2}(w+w^{-1})$. Then7

#### Proof

The main idea is to establish the identity by comparison of thecoefficients of $t^n$ on both sides of the identity. Since

$$ \Big (\frac{u}{t};q\Big )_kt^k=\sum _{j=0}^k(-1)^{k-j}q^{\left( {\begin{array}{c}k-j\\ 2\end{array}}\right) } \frac{(q;q)_k}{(q;q)_j(q;q)_{k-j}}u^{k-j}t^j, $$

by the terminating *q*-binomial theorem [13, Ex. 1.2(vi)], we see that the first ${\phantom {a}}_3\phi _2$ of (7) isNow, the inner sum over *k* is a ${\phantom {a}}_2\phi _2$ series which can be transformed to a multiple of a ${\phantom {a}}_2\phi _1$ series by Jackson’s transformation in [13, (III.4)], which we restate for convenience in the following form:8valid for |z|<1 for convergence of the series on the right-hand side. We thus apply the $(a,b,c,z)\mapsto {(awq^j,a/w,abq^j,u/a)}$ case of (8) and obtain for the first ${\phantom {a}}_3\phi _2$ series on the left-hand side of (7)Thus, the coefficient of $t^j$ of the first ${\phantom {a}}_3\phi _2$ series on the left-hand side of (7) is

$$ \frac{(\frac{u}{a};q)_\infty }{(uw;q)_\infty }\frac{(aw,bw;q)_j}{(q,ab;q)_j}w^{-j} \sum _{k=0}^\infty \frac{(q^jaw,\frac{a}{w};q)_k}{(q,q^jab;q)_k}a^{-k}u^k. $$

Similarly, by symmetry, the coefficient of $t^{n-j}$ of the second ${\phantom {a}}_3\phi _2$ series on the left-hand side of (7) is

$$ \frac{(\frac{u}{c};q)_\infty }{(\frac{u}{w};q)_\infty } \frac{(\frac{c}{w},\frac{d}{w};q)_{n-j}}{(q,cd;q)_{n-j}}w^{n-j} \sum _{l=0}^\infty \frac{(q^{n-j}\frac{c}{w},cw;q)_l}{(q,q^{n-j}cd;q)_l}c^{-l}u^l. $$

In total, the coefficient of $t^n$ of the product of the two ${\phantom {a}}_3\phi _2$ series on the left-hand side of (7) is

$$\begin{aligned}  &   \frac{(\frac{u}{a},\frac{u}{c};q)_\infty }{(uw,\frac{u}{w};q)_\infty }\sum _{j=0}^n \frac{(aw,bw;q)_j}{(q,ab;q)_j} \frac{(\frac{c}{w},\frac{d}{w};q)_{n-j}}{(q,cd;q)_{n-j}}w^{n-2j}\\  &   \qquad \times \sum _{k,l=0}^\infty \frac{(q^jaw,\frac{a}{w};q)_k}{(q,q^jab;q)_k}\frac{(q^{n-j}\frac{c}{w},cw;q)_l}{(q,q^{n-j}cd;q)_l}\frac{u^{k+l}}{a^kc^l}\\  &   =\frac{(\frac{u}{a},\frac{u}{c};q)_\infty }{(uw,\frac{u}{w};q)_\infty } \sum _{k,l=0}^\infty \frac{(aw,\frac{a}{w};q)_k}{(q,ab;q)_k}\frac{(cw,\frac{c}{w};q)_l}{(q,cd;q)_l}\frac{u^{k+l}}{a^kc^l}\\  &   \qquad \times \sum _{j=0}^n\frac{(q^kaw,bw;q)_j}{(q,q^kab;q)_j} \frac{(q^l\frac{c}{w},\frac{d}{w};q)_{n-j}}{(q,q^lcd;q)_{n-j}}w^{n-2j}. \end{aligned}$$

Now the last sum over *j* is a multiple of an Askey–Wilson polynomial by virtue of (6). We thus obtain the following expression for the coefficient of $t^n$ of the left-hand side of (7):

$$\begin{aligned} \frac{(\frac{u}{a},\frac{u}{c};q)_\infty }{(uw,\frac{u}{w};q)_\infty } \sum _{k,l=0}^\infty \frac{(aw,\frac{a}{w};q)_k}{(q,ab;q)_k}\frac{(cw,\frac{c}{w};q)_l}{(q,cd;q)_l}\frac{u^{k+l}}{a^kc^l} \frac{p_n(x;q^ka,b,q^lc,d|q)}{(q,q^kab,q^lcd;q)_n}, \end{aligned}$$

and the result follows. □

An immediate relevant consequence of Theorem 2.3 is obtained by letting u=t, in which case both ${\phantom {a}}_3\phi _2$ basic hypergeometric series reduce to unity. This gives the following closed form summation formula for a triple sum; or, after writing the Askey–Wilson polynomial as a sum, say, using (6), for a basic hypergeometric *quadruple* sum:

#### Corollary 2.4

Let |q|<1 and $w,t,a,b\in {{\mathbb {C}}^*}$ such that $|t|<\min (|a|,|c|,|w|,|w^{-1}|)$, $x=\frac{1}{2}(w+w^{-1})$. Then

9 $$\begin{aligned} \sum _{n,k,l\ge 0}\frac{t^{n+k+l}}{a^kc^l} \frac{(aw^{\{\!\pm \!\}};q)_k(cw^{\{\!\pm \!\}};q)_l\,p_n(x;q^ka,b,q^lc,d|q)}{(q;q)_k(q;q)_l(q;q)_n(ab;q)_{n+k}(cd;q)_{n+l}} =\frac{(tw^{\{\!\pm \!\}};q)_\infty }{(\frac{t}{a},\frac{t}{c};q)_\infty }. \end{aligned}$$

#### Proof

Simply setting u=t in Theorem 2.3 produces the result. □

#### Remark 2.5

Another interesting consequence of Theorem 2.3 is obtained by letting w=d (equivalently w=b). In this case one of the ${\phantom {a}}_3\phi _2$ basic hypergeometric series becomes unity. Further, in this case the Askey–Wilson polynomial simplifies using [22, (130)]

10 $$\begin{aligned} p_n\big (\textstyle {\frac{1}{2}}(d+d^{-1});a,b,c,d|q\big )=d^{-n}(ad,bd,cd;q)_n. \end{aligned}$$

After this reduction, the sum over *l* in (7) is summable using the nonterminating *q*-binomial theorem (cf. [13, (II.3)]) and the triple sum reduces to a double sum. The final result is given bywhich can be seen to be true since the right-hand side is a multiple of a *q*-Appell $\Phi ^{(1)}$ function [25, (17.4.5)] which can be reduced to a ${\phantom {a}}_3\phi _2$ using [25, (17.11.1)].

## Overview and revisitation of terminating *k*-balanced  summations

Askey–Wilson polynomials are given in terms of a terminating balanced ${\phantom {a}}_4\phi _3$. We would like to exploit the Ismail–Wilson generating function for Askey–Wilson polynomials (5) to obtain new product formulas for nonterminating basic hypergeometric series. (The triple summation in Theorem 2.3, while of independent interest and having led to the discovery of Corollary 2.4, appears to be not suitable for our purpose, due to the shifts of the parameters *a* and *c* of the Askey–Wilson polynomial that appears in the summand.) There exist several well-known terminating ${\phantom {a}}_4\phi _3$ summations. Some of them manifestly involve balanced summations and others may be transformed to a balanced ${\phantom {a}}_4\phi _3$ series and, as such, represent summations for Askey–Wilson polynomials which can be applied. In the following, we inspect various known ${\phantom {a}}_4\phi _3$ summation formulas to determine if they may be exploited as described above.

The summation formula [25, (17.7.7)]valid for |q|<1 and $|q\sqrt{a}|<|bc|$, concerns a summation for a nonterminating well-poised ${\phantom {a}}_4\phi _3$ series (cf. [13] for the notion of a well-poised series), but not one for a balanced ${\phantom {a}}_4\phi _3$ series. However, the ${\phantom {a}}_4\phi _3$ series may be transformed to a terminating balanced sum by letting $c=q^{-n}$ and $b=q^n\sqrt{a}$. Unfortunately, in this case the formula vanishes for all n≥1 and is unity for n=0; so the use of this summation will not lead to an interesting product formula when applied to the Ismail–Wilson generating function for Askey–Wilson polynomials (5).

In [1], Andrews (1976) proved the following balanced ${\phantom {a}}_4\phi _3$ summation, hereby giving a *q*-analogue of the terminating version of Watson’s ${\phantom {a}}_3F_2(1)$ summation [25, (16.4.6)]:11provided $b=q^{-n}$ and *n* is a non-negative integer. Replacing the variable *a* by $q^na$ and written in explicit terms, this formula is12In [29], (12) was used to prove the product formula [29, Theorem 3]valid for |q|<1 and |t|<1. By a standard polynomial argument, Andrews’ summation in (12) is equivalent to the following summation found by Jain [20]:13In fact, (11) holds whenever the series terminates; i.e., instead of $b=q^{-n}$ we can take $c=q^{-2n}$, which yields (13). Now, while (13) concerns a balanced ${\phantom {a}}_4\phi _3$ summation, it cannot be written as an *n*-th order Askey–Wilson polynomial (using any of the representations in Theorem 2.1) with parameters independent from *n*.

The very-well-poised ${\phantom {a}}_8W_7$ appearing in the summation [25, (17.7.8)] (which is a Gasper and Rahman’s *q*-analogue of Watson’s ${\phantom {a}}_3F_2$ sum) can be transformed to a sum of two balanced ${\phantom {a}}_4\phi _3$’s using Bailey’s transformation [25, (17.9.16)]. In most choices of parameters which give a terminating representation for the ${\phantom {a}}_8W_7$ one does not obtain a meaningful result as a single balanced ${\phantom {a}}_4\phi _3$. However, one of choices leads to a balanced ${\phantom {a}}_4\phi _3$ summation formula, namely14which vanishes for *n* odd. From (14) one obtains, after the substitution $(b,c)\mapsto (-q^{1-n}/b,a)$, followed by replacing $q^2$ by *q*, and some simplification, the following summation:15The summation formula (15) is amenable to replacements in the Askey–Wilson polynomials using the terminating basic hypergeometric series representation (4). This summation of a balanced terminating ${\phantom {a}}_4\phi _3$ was first derived by Bailey [4] and is a consequence of a formula due to Jackson [19] (see also Carlitz [8], and [13, Exercise 2.6]). Note that (15) is equivalent to (12) (and (13)), which can be seen by replacing $(a,c)\mapsto (a^2b^2/q,a^2)$ in (12) and then applying Sears’ transformation [25, (17.9.14)]. Therefore, it is seen that Andrews’ *q*-analogue (12) of the terminating version of Watson’s ${\phantom {a}}_3F_2(1)$ summation [25, (16.4.6)] is a special case of Gasper–Rahman’s nonterminating *q*-analogue of Watson’s ${\phantom {a}}_3F_2$ sum [25, (17.7.8)].

The summation formula [25, (17.7.10)]where |q|<1 and |c|<1, Gasper and Rahman’s *q*-analogue of Whipple’s ${\phantom {a}}_3F_2(1)$ summation [25, (16.4.7)], is a very-well-poised ${\phantom {a}}_8W_7$ summation formula. It can be transformed to a sum of two balanced ${\phantom {a}}_4\phi _3$’s using Bailey’s transformation [25, (17.9.16)]. If one chooses (a,b,c,d,e,f)↦(-c,a,q/a,c,-d,-q/d) with $d=q^{-n}$ then the coefficients of one of the balanced ${\phantom {a}}_4\phi _3$’s vanishes and we are left with the following balanced ${\phantom {a}}_4\phi _3$ summation formulawhich is equivalent to [25, (17.7.11)], Andrews’ *q*-analogue of the terminating version of Whipple’s ${\phantom {a}}_3F_2(1)$ summation [25, (16.4.7)], a balanced terminating ${\phantom {a}}_4\phi _3$ summation. The simplified formula is [1, Theorem 2], [25, (17.7.11)],16also given in this form in [13, (II.19)], and which can be more explicitly stated as17The following two summations for balanced ${\phantom {a}}_4\phi _3$ series are given in Bressoud, Ismail and Stanton (2000) [7, (2.1), (2.2)]. However, they are given even earlier in Verma and Jain (1980) [33, (5.3), (5.4)]. The first balanced ${\phantom {a}}_4\phi _3$ summation formula [7, (2.2)], [25, (17.7.12)] (also see [13, Exercise 3.34; $c\mapsto bq^n$]) is given by18which is a *q*-analogue of Bailey’s ${\phantom {a}}_4F_3(1)$ summation. We will use this summation formula to derive Theorem 4.18 in Sect. 4. The balanced ${\phantom {a}}_4\phi _3$ summation formula [25, (17.7.13)], [7, (2.1)]19is another *q*-analogue of Bailey’s ${\phantom {a}}_4F_3(1)$ summation. We will use (19) to recover a result by Nassrallah (80) (see also Remark 4.14). Guo [14] and Wei–Wang [35] derived terminating balanced ${\phantom {a}}_4\phi _3$ summations. Guo’s balanced summations are [14, p. 1040, second identity]20[14, (4.4)]21and [14, (4.6)]22Wei and Wang provided the following balanced summation [35, Corollary 6]23and a number of closely related ones.

### Remark 3.1

On first appearance, one might consider (18)–(23) to be different. However, after replacing $q^2\mapsto q$, all the summations (18)–(23) should be considered to be equivalent when viewed as terminating balanced ${\phantom {a}}_4\phi _3$’s which can be written in terms of Askey–Wilson polynomials, namely with Theorem 3.29 below. Using (2), the summations (18) and (19) produce Theorem 3.29 by replacing $(a,b)\mapsto (q^{-\frac{1}{4}}a,ab)$ and $(a,b)\mapsto (q^\frac{1}{4}a,q^\frac{1}{2}ab)$ respectively. Using (4), the summations (20)– (23) produce Theorem 3.29 by replacing $(a,b)\mapsto (q^{-\frac{1}{4}}a,q^{-\frac{1}{4}}b)$, $(a,b)\mapsto (q^{\frac{1}{4}}a,q^{\frac{1}{4}}a)$, and $(a,b)\mapsto (q^{-\frac{1}{4}}a,q^{-\frac{1}{4}}b)$, $(a,b)\mapsto (q^{\frac{1}{4}}a,q^{\frac{3}{4}}b)$ respectively.

The summation [25, (17.7.14)], F. H. Jackson’s *q*-analogue of Dougall’s ${\phantom {a}}_7F_6(1)$ summation, is a summation of a terminating very-well-poised balanced ${\phantom {a}}_8W_7$. It can be written as a multiple of a balanced ${\phantom {a}}_4\phi _3$ by taking $(a,b,c,d,e,f)\mapsto (a,b,c,d,\frac{q^{n+1}a^2}{bcd},q^{-n})$ in Bailey’s transformation [25, (17.9.15)]. After simplification this reduces towhich is equivalent to the *q*-Pfaff–Saalschütz sum [25, (17.7.4)], namely24This is exactly the summation which is responsible for the evaluation in (10), and as such useful for reducing the Ismail–Wilson generating function for Askey–Wilson polynomials by a suitable specialization of the parameters.

The summation [13, Exercise 2.14 (i); $a\mapsto a^2$]25is indeed a summation for a balanced ${\phantom {a}}_4\phi _3$ series but in order to evaluate an *n*-th order Askey–Wilson polynomial (represented as a ${\phantom {a}}_4\phi _3$ series using one of the representations in Theorem 2.1) using (25) would require to make one or more of the parameters *a*, *b*, *c*, *d*, *w* to depend on *n* (which can not be conveniently done when considering generating functions).

Two further terminating balanced ${\phantom {a}}_4\phi _3$ summations are listed in Gasper and Rahman’s textbook. There is [13, (3.10.9); $a\mapsto a^2$ and $w\mapsto abq^{1-n}$]26and also [13, (3.10.10); a↦ab]27The last three summations can actually be easily proved by application of the Sears transformation [13, (3.2.1)] after which the transformed sum, having $q^{-1}$ as an upper parameter, reduces to the sum of its first two terms. The transformation (27) can be seen to be equivalent to (26) by first interchanging a↔b, replacing a↦qa followed by (a,b)↦(1/(qa),1/b) and then replacing z↦1/z.

Further, there are [34, (3.7)]28and [34, (3.8)]29which cannot be written as *n*-th order Askey–Wilson polynomials with parameters independent from *n*. The summation in (28) can be obtained by a careful (c→1) limit from the following ${\phantom {a}}_4\phi _3$ transformation of Berkovich and Warnaar [6, (3.10)]:30Specifically, the limit c→1 in (30) using

$$\begin{aligned}  &   \lim _{c\rightarrow 1}\frac{(c^2;q^2)_k}{(c^2;q)_k}={\left\{ \begin{array}{ll}1& \text {if } k=0 \text {,}\\ \frac{1}{2}(-1;q)_k\qquad & \text {if } k>0 ,\end{array}\right. } \end{aligned}$$

reduces the sum on the left-hand side of the transformation which can then be computed by the *q*-Pfaff–Saalschütz summation [25, (17.7.4)], boiling down, after replacing *b* by *a*/*b*, to (28). (This derivation of (28) from (30) was kindly communicated to us by S. Ole Warnaar in a private communication.)

Different types of balanced and 2-balanced summations were derived by Andrews  [2] and by Guo [14]. The involved sums have the special feature that they are symmetric or almost symmetric with respect to reversing them. We list a few of them as samples. In [2, Theorem 3] Andrews proved the following 2-balanced ${\phantom {a}}_4\phi _3$ summation:31Andrews further made the following related conjecture [2, (6.1)] (again concerning a 2-balanced summation) that was settled by Guo [14, Sect. 3]32Both of the above 2-balanced ${\phantom {a}}_4\phi _3$ summations conjectured by Andrews were also proved in Ismail and Rahman [16], but the statement and proof of (32) therein both contain typographical errors.

### New terminating 1-balanced  summations

While carrying out a systematic search in the literature for terminating balanced ${\phantom {a}}_4\phi _3$ summations, and making computer experiments, we found the following additional summations that are similar to (12), which appear be new. First we consider summations of terminating (1-)balanced ${\phantom {a}}_4\phi _3$ series.

#### Theorem 3.2

Let $q,a,c\in {{\mathbb {C}}^*}$. Then one has the following two equivalent *q*-quadratic summations for a terminating balanced ${\phantom {a}}_4\phi _3$, namely3334where the two summations in (33) and (34) can be shown to be equivalent using Sears’ balanced terminating ${\phantom {a}}_4\phi _3$ transformation [25, (17.9.14)].

#### Proof

We only need to show (33) which we do by induction on *n*. For n=0 the summation in (33) is trivial. We assume that the formula is true up to a fixed non-negative integer *n*. To prove (33) for *n* replaced by n+1, we use the elementary identity

35 $$\begin{aligned} \frac{(1-q^{-n-1})(1-q^{n+k}a)}{(1-q^{-n-1+k})(1-q^{n}a)} =1-q^{-n-1}\frac{(1-q^k)(1-q^{2n+1}a)}{(1-q^{-n-1+k})(1-q^{n}a)} \end{aligned}$$

to obtainThe first ${\phantom {a}}_4\phi _3$ series can be simplified by the inductive hypothesis, whereas the second series can be evaluated by (12), and, in particular, vanishes for odd *n*. As a result, we obtain for the last expression,

$$\begin{aligned}  &   c^{\lfloor \frac{n+1}{2}\rfloor } \frac{(q,\frac{a}{c};q^2)_{\lfloor \frac{n+1}{2}\rfloor }}{(a,qc;q^2)_{\lfloor \frac{n+1}{2}\rfloor }} \end{aligned}$$

in case *n* is odd, and

$$\begin{aligned}&c^{\lfloor \frac{n+1}{2}\rfloor } \frac{(q,\frac{a}{c};q^2)_{\lfloor \frac{n+1}{2}\rfloor }}{(a,qc;q^2)_{\lfloor \frac{n+1}{2}\rfloor }}- q^{-n}\frac{(1-c)(1-q^{2n+1}a)}{(1-qc)(1-a)} c^{\lfloor \frac{n+1}{2}\rfloor } \frac{(q,\frac{a}{c};q^2)_{\lfloor \frac{n+1}{2}\rfloor }}{(q^2a,q^3c;q^2)_{\lfloor \frac{n+1}{2}\rfloor }}\\&=[(1-q^{n+1}c)(1-q^na)-(1-c)(1-q^{2n+1}a)] c^{\lfloor \frac{n+1}{2}\rfloor } \frac{(q,\frac{a}{c};q^2)_{\lfloor \frac{n+1}{2}\rfloor }}{(a,qc;q^2)_{\lfloor \frac{n+1}{2}+1\rfloor }}\\&=c(1-q^{n+1})(1-\frac{q^na}{c})c^{\lfloor \frac{n+1}{2}\rfloor } \frac{(q,\frac{a}{c};q^2)_{\lfloor \frac{n+1}{2}\rfloor }}{(a,qc;q^2)_{\lfloor \frac{n+1}{2}+1\rfloor }} =c^{\lfloor \frac{n+1}{2}+1\rfloor } \frac{(q,\frac{a}{c};q^2)_{\lfloor \frac{n+1}{2}+1\rfloor }}{(a,qc;q^2)_{\lfloor \frac{n+1}{2}+1\rfloor }} \end{aligned}$$

in case *n* is even, which furnishes the theorem. □

#### Theorem 3.3

Let $q,a,c\in {{\mathbb {C}}^*}$. Then one has the following *q*-quadratic summation for a terminating balanced ${\phantom {a}}_4\phi _3$, namely36

#### Proof

This result readily follows from taking $(a,b,c)\mapsto (-\sqrt{a},q^{n}a\sqrt{c})$ in (30). In this case the ${\phantom {a}}_4\phi _3$ series on the right-hand side can be seen to reduce to the serieswhich factorizes in closed form due to the *q*-Pfaff–Saalschütz summation (24). Simplification of the combined factors gives (36). □

The following terminating balanced ${\phantom {a}}_4\phi _3$ is not new but due to Wei et al. [36, Corollary 5], nevertheless we display it in this section, together with a direct proof.

#### Theorem 3.4

Let $q,a,c\in {{\mathbb {C}}^*}$. Then one has the following *q*-quadratic summation for a terminating balanced ${\phantom {a}}_4\phi _3$, namely37

#### Proof

This immediately follows from the $(\sqrt{a},\sqrt{c})\mapsto (-\frac{q^{-n}}{\sqrt{c}},-\frac{q^{-n}}{\sqrt{a}})$ substitution of Theorem 3.3, together with reversing the order of summation of the ${\phantom {a}}_4\phi _3$ series according to [13, Exercise 1.4 (ii)]. □

#### Theorem 3.5

Let $q,a,c\in {{\mathbb {C}}^*}$. Then one has the following two equivalent *q*-quadratic summations for a terminating balanced ${\phantom {a}}_4\phi _3$, namely3839where the two summations in (38) and (39) can be shown to be equivalent using Sears’ balanced terminating ${\phantom {a}}_4\phi _3$ transformation [25, (17.9.14)].

#### Proof

We only need to show (38) which we achieve by the use of contiguous relations. In particular, application of [23, (3.3)] (which is a straightforward extension of [13, Exercise 1.9 (iii)]) yieldsNow the two ${\phantom {a}}_4\phi _3$ series on the right-hand side can be evaluated by different instances of (36). The two terms can be combined to the right-hand side in (38). □

#### Theorem 3.6

Let $q,a,c\in {{\mathbb {C}}^*}$. Then one has the following *q*-quadratic summation for a terminating balanced ${\phantom {a}}_4\phi _3$, namely40

#### Proof

Just as in the proof of Theorem 3.5, we use the contiguous relation [23, (3.3)]. This yieldsNow the two ${\phantom {a}}_4\phi _3$ series on the right-hand side can be evaluated by (33) and (36), respectively. The two terms can be combined to the right-hand side in (40). □

#### Theorem 3.7

Let $q,a,c\in {{\mathbb {C}}^*}$. Then one has the following two equivalent *q*-quadratic summations for a terminating balanced ${\phantom {a}}_4\phi _3$, namely4142where the two summations in (41) and (42) can be shown to be equivalent using Sears’ balanced terminating ${\phantom {a}}_4\phi _3$ transformation [25, (17.9.14)].

#### Proof

It suffices to prove (41). Just as in the proofs of Theorems 3.5 and 3.6, we use the contiguous relation [23, (3.3)]. This yieldsNow the two ${\phantom {a}}_4\phi _3$ series on the right-hand side can be evaluated by different instances of (33). The two terms can be combined to the right-hand side in (41). □

The next balanced ${\phantom {a}}_4\phi _3$ summation does not factorize completely (in particular not for even *n*), but possesses a complete product evaluation for odd *n*.

#### Theorem 3.8

Let $q,a,c\in {{\mathbb {C}}^*}$. Then one has the following two equivalent *q*-quadratic summations for a terminating balanced ${\phantom {a}}_4\phi _3$, namely4344where the two summations in (43) and (44) can be shown to be equivalent using Sears’ balanced terminating ${\phantom {a}}_4\phi _3$ transformation [25, (17.9.14)].

#### Proof

The proof is similar to that of Theorem 3.2 and uses induction on *n*. It suffices to prove (43). Already the n=0 is more involved than that of Theorem 3.2 but still trivial. Using the a↦qa case of (35), the n↦n+1 case of the sum is split into two terms. The first term is simplified by the inductive hypothesis (where division into two cases has to be done, according to the parity of *n*) while the second term is simplified by an instance of Theorem 3.2. The computational details are routine and thus omitted. □

The next two balanced ${\phantom {a}}_4\phi _3$ summations are similar in nature to Theorem 3.8; they possess complete product evaluations for odd *n*.

#### Theorem 3.9

Let $q,a,c\in {{\mathbb {C}}^*}$. Then one has the following two equivalent *q*-quadratic summations for a terminating balanced ${\phantom {a}}_4\phi _3$, namely4546where the two summations in (45) and (46) can be shown to be equivalent using Sears’ balanced terminating ${\phantom {a}}_4\phi _3$ transformation [25, (17.9.14)].

#### Proof

It suffices to prove (45). Just as in the proofs of Theorems 3.5 and 3.6, we use the contiguous relation [23, (3.3)]. This yieldsNow the two ${\phantom {a}}_4\phi _3$ series on the right-hand side can be evaluated by (43) and (38), respectively. The two terms can be combined to the right-hand side in (45). □

#### Theorem 3.10

Let $q,a,c\in {{\mathbb {C}}^*}$. Then one has the following two equivalent *q*-quadratic summations for a terminating balanced ${\phantom {a}}_4\phi _3$, namely4748where the two summations in (47) and (48) can be shown to be equivalent using Sears’ balanced terminating ${\phantom {a}}_4\phi _3$ transformation [25, (17.9.14)].

#### Proof

It suffices to prove (48). Again we use the contiguous relation [23, (3.3)] (but now in the other direction!). This yieldsNow the two ${\phantom {a}}_4\phi _3$ series on the right-hand side can be evaluated by (38) and, inductively, by an instance of (48), respectively. The two terms can be combined to the right-hand side in (48). □

#### Remark 3.11

Since all summations given in this section are given in terms of balanced ${\phantom {a}}_4\phi _3$’s they have corresponding representations in terms of Askey–Wilson polynomials (see Theorem 2.1). When these summations are written in terms of Askey–Wilson polynomials one may be able to ascertain that certain of these summations are equivalent. In particular, Theorems 3.9 and 3.10, can be seen to be equivalent summations when viewed in terms of their representations in terms of Askey–Wilson polynomials. For instance if one uses the first part of Theorem 3.9, makes the replacement $(a,c)\mapsto (a^2b^2,q^{-1}b^2)$ followed by replacing (a,b)↦(-a,-b) then one finds that the parameters for the corresponding Askey–Wilson polynomials are $(w,a,b,c,d)=(iq^{\frac{1}{2}},ib,-iqb,-iqa,ia)$. For instance, if one uses the first part of Theorem 3.10, makes the replacement $(a,c)\mapsto (a^2b^2,qb^2)$ then one finds that the parameters of the corresponding Askey–Wilson polynomials are $(w,a,b,c,d)=(iq^{\frac{1}{2}},-iqb,ib,ia,-iqa)$. Because the Askey–Wilson polynomials are invariant under *a*, *b*, *c*, *d* parameter interchange, we see that both of these summation theorems lead to the same summation formula for Askey–Wilson polynomials, namely Theorem 3.27 below. So in this sense, both Theorems 3.9 and 3.10 can be considered to be equivalent. In the same sense, one can see that Theorem 3.6 is also equivalent to Theorem 3.7.

### New terminating 2-balanced and 3-balanced  summations

The following terminating 2-balanced ${\phantom {a}}_4\phi _3$ summation is not new but due to Wei et al. [36, Corollary 2], nevertheless we display it in this section, together with a direct proof.

#### Theorem 3.12

Let $q,a,c\in {{\mathbb {C}}^*}$. Then one has the following *q*-quadratic summation for a terminating 2-balanced ${\phantom {a}}_4\phi _3$, namely49

#### Proof

This result can be proved by induction, using the simple decomposition

50 $$\begin{aligned} \frac{(1-c)}{(1-q^kc)}=1-c\frac{(1-q^k)}{(1-q^kc)} \end{aligned}$$

and the balanced ${\phantom {a}}_4\phi _3$ summation in (12). The details are routine and are omitted. □

We now provide two new 2-balanced ${\phantom {a}}_4\phi _3$ summations.

#### Theorem 3.13

Let $q,a,c\in {{\mathbb {C}}^*}$. Then one has the following *q*-quadratic summation for a terminating 2-balanced ${\phantom {a}}_4\phi _3$, namely51

#### Proof

The proof is similar to that of Theorem 3.12. One proceeds by induction and uses the c↦c/q case of (50), together with the inductive hypothesis and the $c\mapsto c/q^2$ instance of (34). □

#### Theorem 3.14

Let $q,a,c\in {{\mathbb {C}}^*}$. Then one has the following *q*-quadratic summation for a terminating 2-balanced ${\phantom {a}}_4\phi _3$, namely52

#### Proof

The proof uses induction, the $c\mapsto q^{n-1}a$ case of (50), the inductive hypothesis and (33). □

Similar to (43), the summation in (52) can be viewed as esoteric.

Further, we have derived two new 3-balanced terminating ${\phantom {a}}_4\phi _3$ summations. The following summation is somewhat esoteric but possesses a complete product evaluation for odd *n*.

#### Theorem 3.15

Let $q,a,c\in {{\mathbb {C}}^*}$. Then one has the following *q*-quadratic summation for a terminating 3-balanced ${\phantom {a}}_4\phi _3$, namely53

#### Proof

The proof uses induction, the $c\mapsto q^{n-1}a$ case of (50), the inductive hypothesis and Theorem 3.13. □

Some further *k*-balanced ${\phantom {a}}_4\phi _3$ summations, closely related (more specifically, contiguous) to the ones in the subsection, are given by Wei, Gong and Li in [36, Corollaries 3 and 6]. The ${\phantom {a}}_4\phi _3$ summations in this subsection that do not completely factorize (typically depending on the parity of *n*) can be specialized such that the right-hand sides do factorize into closed form. There may be different ways to achieve this. We conclude this subsection with two such examples. The following summation for a terminating 2-balanced ${\phantom {a}}_4\phi _3$ series arises from Theorem 3.14 directly by the specialization $a\mapsto -q^{1-2n}$. It is easy to see that in this case the right-hand side completely factorizes into closed form without division into cases.

#### Corollary 3.16

Let $q,c\in {{\mathbb {C}}^*}$. Then one has the following *q*-quadratic summation for a terminating 2-balanced ${\phantom {a}}_4\phi _3$, namely54

The following summation for a terminating 3-balanced ${\phantom {a}}_4\phi _3$ series arises from Theorem 3.15 directly by the specialization $a\mapsto -q^{1-2n}$. Also in this case, the right-hand side completely factorizes into closed form without division into cases.

#### Corollary 3.17

Let $q,c\in {{\mathbb {C}}^*}$. Then one has the following *q*-quadratic summation for a terminating 3-balanced ${\phantom {a}}_4\phi _3$, namely55

#### Remark 3.18

We believe that to find direct proofs of Corollaries 3.16 and 3.17, say by induction, would be quite a challenge, even though terminating summations such as the summations in (54) and (55) can principally be proved in an automatic fashion using computer algebra with implementations of well-known algorithms (and their *q*-analogues), such as those due to Gosper, Zeilberger, and Petkovšek (cf. [27, 28]).

### *q*-Quadratic special values for Askey–Wilson polynomials

We now derive ten *q*-quadratic special values for Askey–Wilson polynomials $p_n(x;a,b,c,d|q)$: (1) 5 with argument x=0; (2) 3 with argument $x=\frac{\mathrm i}{2}(q^\frac{1}{2}-q^{-\frac{1}{2}})$; (3) 1 with argument $x=\frac{1}{2}(q^\frac{1}{4}+q^{-\frac{1}{4}})$; and (4) 1 with argument $x=\frac{1}{2}(qa+(qa)^{-1})$.

#### Remark 3.19

Note that we arrange the following special values such that closed-form representations are given separately for *n* even with *q*-shifted factorial index and powers given as $\tfrac{1}{2} n$; and *n* odd with *q*-shifted factorial index and powers given as $\tfrac{1}{2}(n-1)$. This way, when these special values are applied to linear and bilinear generating functions such as the Ismail–Wilson generating function, after one splits the series into contributions over *n* even and *n* odd, one is already in a form suitable to derive nonterminating basic hypergeometric representations which correspond to parity values.

Corresponding to Bailey’s summation (15) (and equivalently (12), (13)), one has the following *q*-quadratic special value for Askey–Wilson polynomials.

#### Theorem 3.20

Let $$n\in \mathbb {N}_0$$, $$q,a,b\in {{\mathbb {C}}^*}$$. Then

56 $$\begin{aligned}  &   p_n(0;\{\!\pm \!\}{\mathrm i}a,\{\!\pm \!\}{\mathrm i}b|q)= \left\{ \begin{array}{ll} \displaystyle (-1)^{\frac{n}{2}}\frac{(q,a^2,b^2,\{\!\pm \!\}ab,\{\!\pm \!\}qab;q^2)_{\frac{n}{2}}}{(a^2b^2;q^2)_{\frac{n}{2}}} &  \ \textrm{if}\ n\ \textrm{even},\\ \displaystyle 0 &  \ \textrm{if}\ n\ \textrm{odd}. \end{array} \right. \nonumber \\ \end{aligned}$$

#### Proof

Starting with (15) then comparing with (3) with $$(w,a,b,c,d)\mapsto (-{\mathrm i},{\mathrm i}a,{\mathrm i}b,$$$$-{\mathrm i}a,-{\mathrm i}b)$$, then replacing $${\mathrm i}\mapsto -{\mathrm i}$$. Let $$m\in \mathbb {N}_0$$. Finally replacing n=2m for *n* even, and n=2m+1 for *n* odd, followed by applying standard identities for *q*-shifted factorials, and solving for *m* in terms of *n* completes the proof. □

Corresponding to our new balanced ${\phantom {a}}_4\phi _3$ summation given in Theorem 3.2 we derive the following *q*-quadratic special value for Askey–Wilson polynomials.

#### Theorem 3.21

Let $n\in \mathbb {N}_0$, $q,a,b\in {{\mathbb {C}}^*}$. Then the Askey–Wilson polynomials have the following *q*-quadratic special value,

57 $$\begin{aligned}  &   p_n(0;\{\!\pm \!\}{\mathrm i}a,{\mathrm i}b,-{\mathrm i}qb|q)\nonumber \\  &   \quad \qquad =(-{\mathrm i})^nb^{2\lfloor \frac{n+1}{2}\rfloor -n} \frac{(qb^2,\{\!\pm \!\}ab;q)_n(q,a^2;q^2)_{\lfloor \frac{n+1}{2}\rfloor }}{(qb^2,a^2b^2;q^2)_{\lfloor \frac{n+1}{2}\rfloor }}\nonumber \\  &   \quad \qquad =\left\{ \begin{array}{ll} \displaystyle (-1)^{\frac{n}{2}}\frac{(q,a^2,q^2b^2,\{\!\pm \!\}ab,\{\!\pm \!\}qab;q^2)_{\frac{n}{2}}}{(a^2b^2;q^2)_{\frac{n}{2}}} & \qquad \textrm{if}\ n\ \textrm{even},\\ \displaystyle -{\mathrm i}(-1)^{\frac{n-1}{2}}(1-q)(1-a^2)b& \\ \quad \times \dfrac{(q^3,q^2a^2,q^2b^2,\{\!\pm \!\}qab,\{\!\pm \!\}q^2ab;q^2)_{\frac{n-1}{2}}}{(q^2a^2b^2;q^2)_{\frac{n-1}{2}}} &  \quad \,\,\,~\textrm{if}\ n\ \textrm{odd}. \end{array} \right. \end{aligned}$$

#### Proof

Replace

$$ (a,c)\mapsto (a^2b^2,b^2) $$

in either (33), (34) then setting

$$ (w,a,b,c,d)\mapsto ({\mathrm i},{\mathrm i}a,-{\mathrm i}a,{\mathrm i}b,-{\mathrm i}qb) $$

in (2) produces (57). Then replacing n=2m for *n* even, and n=2m+1 for *n* odd, followed by standard identities for *q*-shifted factorials, finally solving for *m* in terms of *n* completes the proof. □

Corresponding to our new balanced ${\phantom {a}}_4\phi _3$ summation given in Theorem 3.5 we derive the following *q*-quadratic special value for Askey–Wilson polynomials.

#### Theorem 3.22

Let $n\in \mathbb {N}_0$, $q,a,b\in {{\mathbb {C}}^*}$. Then the Askey–Wilson polynomials have the following *q*-quadratic special value,

58 $$\begin{aligned}  &   p_n(0;{\mathrm i}a,-{\mathrm i}qa,{\mathrm i}b,-{\mathrm i}qb|q)\nonumber \\  &   \quad = \left\{ \begin{array}{ll} \displaystyle (-1)^{\frac{n}{2}}\frac{(q,q^2a^2,q^2b^2,qab,-ab,q^2ab,-qab;q^2)_{\frac{n}{2}}}{(q^2a^2b^2;q^2)_{\frac{n}{2}}} &  \qquad \textrm{if}\ n\ \textrm{even}, \\ \displaystyle -{\mathrm i}(-1)^{\frac{n-1}{2}}(1-q)(a+b)(1-qab)&  \\ \displaystyle \quad \times \frac{(q^3,q^2a^2,q^2b^2,q^2ab,-qab,q^3ab,-q^2ab;q^2)_{\frac{n-1}{2}}}{(q^2a^2b^2;q^2)_{\frac{n-1}{2}}}& \qquad ~\textrm{if}\ n\ \textrm{odd}. \end{array} \right. \end{aligned}$$

#### Proof

Replace

$$ (a,c)\mapsto (a^2b^2,a^2) $$

then replace (a,b)↦(-a,-b) in either (38), (39) then setting

$$ (w,a,b,c,d)\mapsto ({\mathrm i},{\mathrm i}qa,-{\mathrm i}a,{\mathrm i}qb,-{\mathrm i}b) $$

in (2) and replacing n=2m for *n* even, and n=2m+1 for *n* odd, followed by standard identities for *q*-shifted factorials, finally solving for *m* in terms of *n* completes the proof. □

Corresponding to Andrews’ *q*-analogue of the terminating version of Whipple’s ${\phantom {a}}_3F_2(1)$ summation (17) (cf. [25, (16.4.7)]), one has the following *q*-quadratic special value for Askey–Wilson polynomials.

#### Theorem 3.23

Let $$n\in \mathbb {N}_0$$, $$q,a,b\in {{\mathbb {C}}^*}$$. Then

59 $$\begin{aligned}  &   p_n(0;{\mathrm i}a,\tfrac{{\mathrm i}q}{a},{\mathrm i}b,\tfrac{{\mathrm i}q}{b}|q)\nonumber \\  &   \quad =\left\{ \begin{array}{ll} \displaystyle (-1)^{\frac{n}{2}}(-q,-q^2,-ab,-\tfrac{q^2}{ab},-\tfrac{qa}{b},-\tfrac{qb}{a};q^2)_{\frac{n}{2}} &  \ \textrm{if}\ n\ \textrm{even},\\ \displaystyle -\tfrac{{\mathrm i}q}{b}(-1)^{\frac{n-1}{2}}(1+q)(1+\tfrac{ab}{q})(1+\tfrac{b}{a})&  \\ \quad \times (-q^2,-q^3,-qab,-\tfrac{q^3}{ab},-\tfrac{q^2a}{b},-\tfrac{q^2b}{a};q^2)_{\frac{n-1}{2}}& \ ~\textrm{if}\ n\ \textrm{odd}. \end{array} \right. \end{aligned}$$

#### Proof

Starting with (17) then comparing with (2) with $(w,a,b,c,d)\mapsto (-{\mathrm i},{\mathrm i}a,{\mathrm i}q/a,$ $-{\mathrm i}b/a,-{\mathrm i}qa/b)$ followed by $i\mapsto -{\mathrm i}$ and b↦ab and b↦-b. Let $m\in \mathbb {N}_0$. Replacing n=2m for *n* even, and n=2m+1 for *n* odd, followed by applying standard identities for *q*-shifted factorials, and solving for *m* in terms of *n* completes the proof. □

Corresponding to our new balanced esoteric ${\phantom {a}}_4\phi _3$ summation given in Theorem 3.8 we derive the following *q*-quadratic special value for Askey–Wilson polynomials.

#### Theorem 3.24

Let $n\in \mathbb {N}_0$, $q,a,b\in {{\mathbb {C}}^*}$. Then the Askey–Wilson polynomials have the following esoteric *q*-quadratic special value,

60 $$\begin{aligned}  &   p_n(0;\{\!\pm \!\}{\mathrm i}a,{\mathrm i}b,-{\mathrm i}q^2b|q)\nonumber \\  &   \quad = \left\{ \begin{array}{ll} \displaystyle \frac{(-1)^{\frac{n}{2}}(a^2,q^2b^2,\{\!\pm \!\}ab,\{\!\pm \!\}qab;q^2)_{\frac{n}{2}}}{(1-q^2b^2)(1-a^2b^2)(q^2a^2b^2;q^2)_{\frac{n}{2}}}&  \\ \displaystyle \hspace{0.25cm}\times \Biggl (\frac{(1-qb^2)(1-qa^2b^2)(q,q^3b^2,q^3a^2b^2;q)_{\frac{n}{2}}}{(qb^2,qa^2b^2;q^2)_{\frac{n}{2}}} \\ \displaystyle \hspace{2.5cm}+\frac{qb^2(1-q)(1-\frac{a^2}{q})(q^3,qa^2;q^2)_{\frac{n}{2}}}{(\frac{a^2}{q};q^2)_{\frac{n}{2}}}\Biggr ) &  \textrm{if}\ n\ \textrm{even}, \\ \displaystyle -{\mathrm i}(-1)^{\frac{n-1}{2}}b(1-q^2)(1-a^2) &  \\ \quad \times \dfrac{(q^3,q^2a^2,q^4b^2,\{\!\pm \!\}qab,\{\!\pm \!\}q^2ab;q^2)_{\frac{n-1}{2}}}{(q^2a^2b^2;q^2)_{\frac{n-1}{2}}}&  \textrm{if}\ n\ \textrm{odd}. \end{array} \right. \end{aligned}$$

#### Proof

Replacing $(a,c)\mapsto (a^2b^2,a^2)$ in (43) and then comparing with (2) using $(w,a,b,c,d)=({\mathrm i},\{\!\pm \!\}{\mathrm i}a,{\mathrm i}b,-{\mathrm i}q^2b)$. Replacing n=2m for *n* even, and n=2m+1 for *n* odd, followed by applying standard identities for *q*-shifted factorials, and solving for *m* in terms of *n* completes the proof. □

Corresponding to our new balanced ${\phantom {a}}_4\phi _3$ summation given in Theorem 3.4 we derive the following *q*-quadratic special value for Askey–Wilson polynomials with argument on the imaginary axis.

#### Theorem 3.25

Let $n\in \mathbb {N}_0$, $q,a,b\in {{\mathbb {C}}^*}$. Then the Askey–Wilson polynomials have the following *q*-quadratic special value,

61 $$\begin{aligned}  &   p_n(\tfrac{\mathrm i}{2}(q^\frac{1}{2}-q^{-\frac{1}{2}});\{\!\pm \!\}{\mathrm i}a,\{\!\pm \!\}{\mathrm i}b|q)\nonumber \\  &   \quad =q^{-\frac{n}{2}} \left\{ \begin{array}{ll} \displaystyle (-1)^{\frac{n}{2}}\frac{(q,qa^2,qb^2,\{\!\pm \!\}ab,\{\!\pm \!\}qab;q^2)_{\frac{n}{2}}}{(a^2b^2;q^2)_{\frac{n}{2}}} &  \qquad \textrm{if}\ n\ \textrm{even}, \\ \displaystyle -{\mathrm i}(-1)^{\frac{n-1}{2}}(1-q)(1-a^2b^2) &  \\ \quad \qquad \times \dfrac{(q^3,qa^2,qb^2,\{\!\pm \!\}qab,\{\!\pm \!\}q^2ab;q^2)_{\frac{n-1}{2}}}{(a^2b^2;q^2)_{\frac{n-1}{2}}}&  \qquad ~\textrm{if}\ n\ \textrm{odd}. \end{array} \right. \end{aligned}$$

#### Proof

Replace

$$ (a,c)\mapsto (a^2b^2/q^2,a^2/q) $$

in (37) then setting

$$ (w,a,b,c,d)\mapsto ({\mathrm i}q^{\frac{1}{2}},\{\!\pm \!\}{\mathrm i}a,\{\!\pm \!\}{\mathrm i}b) $$

in (2) and replacing n=2m for *n* even, and n=2m+1 for *n* odd, followed by standard identities for *q*-shifted factorials, finally solving for *m* in terms of *n* completes the proof. □

Corresponding to our new balanced ${\phantom {a}}_4\phi _3$ summation given in Theorem 3.6 we derive the following *q*-quadratic special value for Askey–Wilson polynomials with argument on the imaginary axis.

#### Theorem 3.26

Let $n\in \mathbb {N}_0$, $q,a,b\in {{\mathbb {C}}^*}$. Then the Askey–Wilson polynomials have the following *q*-quadratic special value,

62 $$\begin{aligned}  &   p_n(\tfrac{\mathrm i}{2}(q^\frac{1}{2}-q^{-\frac{1}{2}});\{\!\pm \!\}{\mathrm i}a,{\mathrm i}b,-{\mathrm i}qb|q)=q^{-\frac{n}{2}}\nonumber \\  &   \quad \times \left\{ \begin{array}{ll} \displaystyle (-1)^{\frac{n}{2}}\frac{(q,qa^2,q^{\frac{5}{2}}b,qb^2,\{\!\pm \!\}ab,\{\!\pm \!\}qab;q^2)_{\frac{n}{2}}}{(q^{\frac{1}{2}}b,a^2b^2;q^2)_{\frac{n}{2}}} &  \quad \textrm{if}\ n\ \textrm{even}, \\ \displaystyle -{\mathrm i}(-1)^{\frac{n-1}{2}}(1-q)(1+q^{\frac{1}{2}}b)(1-q^{\frac{1}{2}}a^2b) &  \\ \qquad \qquad \displaystyle \times \frac{(q^3,qa^2,q^3b^2,\{\!\pm \!\}qab,\{\!\pm \!\}q^2ab,q^{\frac{5}{2}}a^2b;q^2)_{\frac{n-1}{2}}}{(q^\frac{1}{2}a^2b,q^2a^2b^2;q^2)_{\frac{n-1}{2}}}&  \quad ~\textrm{if}\ n\ \textrm{odd}. \end{array} \right. \nonumber \\ \end{aligned}$$

#### Proof

Replace

$$ (a,c)\mapsto (a^2b^2,a^2/q) $$

followed by b↦-b in (40) then setting

$$ (w,a,b,c,d)\mapsto ({\mathrm i}q^{\frac{1}{2}},\{\!\pm \!\}{\mathrm i}a,{\mathrm i}b,-{\mathrm i}qb) $$

in (2) and replacing n=2m for *n* even, and n=2m+1 for *n* odd, followed by standard identities for *q*-shifted factorials, finally solving for *m* in terms of *n* completes the proof. □

One has the following *q*-quadratic special value for Askey–Wilson polynomials which corresponds to Theorem 3.9.

#### Theorem 3.27

Let $n\in \mathbb {N}_0$, $q,a,b\in {{\mathbb {C}}^*}$. Then the Askey–Wilson polynomials have the following special value,

63 $$\begin{aligned}  &   p_n(\tfrac{\mathrm i}{2}(q^\frac{1}{2}-q^{-\frac{1}{2}});{\mathrm i}a,-{\mathrm i}qa,{\mathrm i}b,-{\mathrm i}qb|q)\nonumber \\  &   \quad = \left\{ \begin{array}{ll} \displaystyle \frac{(-1)^{\frac{n}{2}}(q,qa^2,qb^2,-ab,\{\!\pm \!\}qab,q^2ab;q^2)_{\frac{n}{2}}}{q^{\frac{n}{2}+1}(1+ab)(1-\frac{1}{q^{\frac{1}{2}}a})(q^2a^2b^2;q^2)_{\frac{n}{2}}} &  \\ \displaystyle \quad \times \Biggl (\frac{(1-\frac{q^{\frac{1}{2}}}{a})(1+qab)(q^\frac{5}{2}b,-q^3ab;q^2)_{\frac{n}{2}}}{(q^\frac{1}{2}b,-qab;q^2)_{\frac{n}{2}}}& \\ \displaystyle \qquad -\frac{(1-q)(1+q^\frac{1}{2}b)(q^3,q^3b^2;q^2)_{\frac{n}{2}}}{(q,qb^2;q^2)_{\frac{n}{2}}}\Biggr ) & \quad \textrm{if}\ n\ \textrm{even}, \\ \displaystyle -{\mathrm i}q^{-\frac{n}{2}}(-1)^{\frac{n-1}{2}}(1-q)(1+q^\frac{1}{2}a)(1+q^\frac{1}{2}b)(1-qab)\\ \displaystyle \quad \times \frac{(q^3,q^3a^2,q^3b^2,-qab,\{\!\pm \!\}q^2ab,q^3ab;q^2)_{\frac{n-1}{2}}}{(q^2a^2b^2;q^2)_{\frac{n-1}{2}}}&  \quad ~\textrm{if}\ n\ \textrm{odd}. \end{array} \right. \end{aligned}$$

#### Proof

Start for instance with the first part of Theorem 3.9, identify the parameters of the Askey–Wilson polynomials using (2) and scale the parameters appropriately. Splitting into even and odd parts completes the proof. □

One has the following special value for the Askey–Wilson polynomials which follows from the equivalent summations (26), (27).

#### Theorem 3.28

Let $n\in \mathbb {N}_0$, $q,a,b\in {{\mathbb {C}}^*}$. Then the Askey–Wilson polynomials have the following *q*-quadratic special value,

64 $$\begin{aligned}  &   p_n(\tfrac{1}{2}(qa+\tfrac{1}{qa});\{\!\pm \!\}q^\frac{1}{2},a,b|q)\nonumber \\  &   \quad =(qa)^{-n} \left\{ \begin{array}{ll} \displaystyle \frac{(\{\!\pm \!\}q^\frac{1}{2}a,\{\!\pm \!\}q^\frac{3}{2}a,ab,qab,\{\!\pm \!\}q^2\sqrt{ab};q^2)_{\frac{n}{2}}}{(\{\!\pm \!\}\sqrt{ab};q^2)_{\frac{n}{2}}} &  \quad \textrm{if}\ n\ \textrm{even}, \\ (1-qa^2)(1-q^2ab)& \\ \displaystyle \quad \times \frac{(\{\!\pm \!\}q^\frac{3}{2}a,\{\!\pm \!\}q^\frac{5}{2}a,qab,q^2ab,\{\!\pm \!\}q^3\sqrt{ab};q^2)_{\frac{n-1}{2}}}{(\{\!\pm \!\}q\sqrt{ab};q^2)_{\frac{n-1}{2}}} & \quad ~\textrm{if}\ n\ \textrm{odd}. \end{array} \right. \end{aligned}$$

#### Proof

Start with (26) and convert to an Askey–Wilson polynomial using (4). Breaking the right-hand side into even and odd parts and shifting the parameters accordingly completes the proof. □

One has the following special value for the Askey–Wilson polynomials which follows directly from the equivalent balanced summations (18)–(23) (see Remark 3.1).

#### Theorem 3.29

Let $n\in \mathbb {N}_0$, $q,a,b\in {{\mathbb {C}}^*}$. Then the Askey–Wilson polynomials have the following special value,

65 $$\begin{aligned}  &   p_n(\tfrac{1}{2}(q^\frac{1}{4}+q^{-\frac{1}{4}});a,q^{\frac{1}{2}}a,b,q^{\frac{1}{2}}b|q)=q^{-\frac{n}{4}}\nonumber \\  &   \quad \times \left\{ \begin{array}{ll} \displaystyle (q^\frac{1}{2}a^2,q^\frac{3}{2}a^2,ab,q^\frac{1}{2}ab,qab,q^\frac{3}{2}ab ;q^2)_{\frac{n}{2}} &  \\ \quad \times \displaystyle \frac{(-q^{\frac{1}{2}},-q,q^{\frac{1}{4}}b,q^{\frac{3}{4}}b;q)_{\frac{n}{2}}}{(-q^{\frac{1}{4}}a,-q^{\frac{3}{4}}a,ab,q^{\frac{1}{2}}ab;q)_{\frac{n}{2}}}& \quad \textrm{if}\ n\ \textrm{even}, \\ \displaystyle (1+q^\frac{1}{2})(1-q^\frac{1}{4}a)(1-q^{\frac{1}{4}}b)(1-q^{\frac{1}{2}}ab)& \\ \quad \displaystyle \times (q^\frac{3}{2}a^2,q^\frac{5}{2}a^2,qab,q^\frac{3}{2}ab,q^2ab,q^\frac{5}{2}ab ;q^2)_{\frac{n-1}{2}}&  \\ \quad \times \displaystyle \frac{(-q,-q^{\frac{3}{2}},q^{\frac{3}{4}}b,q^{\frac{5}{4}}b;q)_{\frac{n-1}{2}}}{(-q^{\frac{3}{4}}a,-q^{\frac{5}{4}}a,q^{\frac{1}{2}}ab,qab;q)_{\frac{n-1}{2}}}& \quad ~\textrm{if}\ n\ \textrm{odd}. \end{array} \right. \end{aligned}$$

#### Proof

Starting with for instance, the Bressoud–Ismail–Stanton summation (18), let $q^2\mapsto q$ and identify the parameters of the Askey–Wilson polynomials using (2). Splitting into even and odd parts completes the proof. □

## Nonterminating product transformations

Starting from our new *q*-quadratic special values of the Askey–Wilson polynomials given in Theorems 3.24 and 3.26, we obtain two 4-term transformation formulas for nonterminating products of two ${\phantom {a}}_2\phi _1$’s and two 3-term transformation formulas. In particular, starting from the esoteric special value of the Askey–Wilson polynomials (60), one obtains the following transformations for nonterminating products of two ${\phantom {a}}_2\phi _1$’s.

### Theorem 4.1

Let 0<|q|<1, $$a,c\in {{\mathbb {C}}^*}$$, |t|<1. Then66

### Proof

Start with the Ismail–Wilson product generating function for Askey–Wilson polynomials (5), and replace $(w,a,b,c,d)\mapsto ({\mathrm i},\{\!\pm \!\}{\mathrm i}a,{\mathrm i}b,-{\mathrm i}q^2b)$ using Theorem 3.24. Finally splitting into even and odd terms using (60) and simplifying completes the proof. □

### Theorem 4.2

Let 0<|q|<1, $$a,b\in {{\mathbb {C}}^*}$$, |t|<1. Then67

### Proof

Start with the Ismail–Wilson product generating function for Askey–Wilson polynomials (5), and replace $(w,a,b,c,d)\mapsto ({\mathrm i},{\mathrm i}a,{\mathrm i}b,-{\mathrm i}a,-{\mathrm i}q^2b)$ using Theorem 3.24. Finally splitting into even and odd parts using (60) and simplifying completes the proof. □

Starting from Theorem 3.22, we obtain the following product transformation formula.

### Theorem 4.3

Let 0<|q|<1, $$a,b\in {{\mathbb {C}}^*}$$, |t|<1. Then68

### Proof

Start with the Ismail–Wilson product generating function for Askey–Wilson polynomials (5), and replace $(w,a,b,c,d)\mapsto ({\mathrm i},{\mathrm i}a,-{\mathrm i}qa,{\mathrm i}b,-{\mathrm i}qb)$ using Theorem 3.22. Finally splitting into even and odd terms using (58) and simplifying completes the proof. □

If one replaces $$\{a,b,t\}\mapsto \{q^a,q^b,\tfrac{1}{2}(1-q^2)z\}$$ in Theorem 4.3, then one obtains the following transformation formula for a product of two confluent hypergeometric functions which is a transformation of type [12, (4.3.5–6), (4.3.13)]. Note that [12, (4.3.13)] contains a typo, the numerator element $-a^{\prime }$ should be $a^{\prime }$.

### Corollary 4.4

Let $$a,b,z\in {{\mathbb {C}}}$$. Then69

### Proof

Start with Theorem 4.3 and make the replacements $\{a,b,z\}\mapsto \{q^a,q^b,\tfrac{1}{2}(1-q^2)z\}$. Then take the $q\rightarrow 1^{-}$ limit and setting t↦z/2 completes the proof. □

If one replaces b↦a then the second term on the right-hand side vanishes and we obtain the following interesting identity70which is a special case of [12, (4.3.5)]. Starting from Theorem 3.26, we obtain the following.

### Theorem 4.5

Let 0<|q|<1, $$a,b\in {{\mathbb {C}}^*}$$, |t|<1. Then71

### Proof

Start with the Ismail–Wilson product generating function for Askey–Wilson polynomials (5), and replace $(w,a,b,c,d)\mapsto ({\mathrm i},\{\!\pm \!\}{\mathrm i}a,{\mathrm i}b,-{\mathrm i}qb)$ using Theorem 3.26. Finally splitting into even and odd terms using (58) and simplifying completes the proof. □

By rearranging the coefficient of the Askey–Wilson polynomial associated with the *q*-quadratic special value used above, then one obtains the following transformation.

### Theorem 4.6

Let 0<|q|<1, $$a,b\in {{\mathbb {C}}^*}$$, |t|<1. Then72

### Proof

Start with the Ismail–Wilson product generating function for Askey–Wilson polynomials (5), and replace $(w,a,b,c,d)\mapsto ({\mathrm i},{\mathrm i}a,{\mathrm i}b,-{\mathrm i}a,-{\mathrm i}qb)$ using Theorem 3.21. Finally splitting into even and odd terms using (58) and simplifying completes the proof. □

By starting with the Ismail–Wilson generating function for Askey–Wilson polynomials (5), substituting parameters and *w* accordingly using Theorem 3.28, we produce the following summation for a nonterminating ${\phantom {a}}_2\phi _1$ which can be expressed as a sum of two nonterminating *q*-quartic ${\phantom {a}}_3\phi _2$’s with quadratic argument.

### Theorem 4.7

Let 0<|q|<1, $a,b,t\in {{\mathbb {C}}^*}$ such that |t|<1. Then73

### Proof

Start with the Ismail–Wilson generating function for Askey–Wilson polynomials (5) and replace

$$ (a,b,c,d)\mapsto (\{\!\pm \!\}q^\frac{1}{2},a,b). $$

and first w=qa and then w=1/(qa). This produces two separate products of ${\phantom {a}}_2\phi _1$’s and in one of them both of them can be summed, one because it is terminating and the other can be written as the *q*-binomial theorem with base $q^2$. In the other, one of the ${\phantom {a}}_2\phi _1$’s is summable using the *q*-binomial theorem with base $q^2$. Then splitting the infinite series into even and odd parts, utilizing Theorem 3.28 one obtains a sum of two ${\phantom {a}}_6\phi _5$’s with base $q^2$ which can then be written as a sum of two ${\phantom {a}}_3\phi _2$’s with base $q^4$. Finally, simplifying, the nonterminating summation follows. □

### Remark 4.8

The first equality in (73) is not deep as the evaluation is easily seen to follow directly from contiguous relations. Indeed in the ${\phantom {a}}_2\phi _1$ series there is a pair of corresponding upper and lower parameters, namely *qab* and *ab*, that multiplicatively only differ by the base *q*. This explains that the left-hand side can be expanded in two terms, each being a summable ${\phantom {a}}_1\phi _0$ series. The two ${\phantom {a}}_3\phi _2$ series also have similar pairs of corresponding contiguous upper and lower parameters. That means that the second expression on the right-hand side essentially consists of 4 terms which are all ${\phantom {a}}_2\phi _1$ series. The second equality is much more interesting than the first, since the just mentioned ${\phantom {a}}_2\phi _1$ series are not summable by the *q*-Gauss summation.

By starting with Theorem 3.29 and utilizing the Ismail–Wilson generating function for Askey–Wilson polynomials we obtain the following transformation formula.

### Theorem 4.9

Let 0<|q|<1, $$a,b\in {{\mathbb {C}}^*}$$, |t|<1. Then74

### Proof

Start with the Ismail–Wilson generating function for Askey–Wilson polynomials (5) and replace

$$ (z,a,b,c,d)\mapsto (q^\frac{1}{4},a,q^\frac{1}{2}a,b,q^\frac{1}{2}b). $$

Then splitting the infinite series into even and odd parts, utilizing Theorem 3.29 and simplifying, the transformation follows. □

This transformation has an interesting $q\rightarrow 1^{-}$ limit75Note that integral representations for the product of two nonterminating ${\phantom {a}}_2\phi _1$’s appearing Theorems 4.5–4.2 can be computed in the same manner as was computed above. However, we leave these as exercises for the reader.

In [29], (16) was used to prove the product formula [29, Theorem 4] (which we reproduce in (76) below). We give an independent proof and are also provide an integral representation for this product.

### Theorem 4.10

Let |q|<1, $f,a,b,z\in {{\mathbb {C}}^*}$ with |z|<1, $w={\mathrm e}^{i\psi }$, $\sigma \in (0,\infty )$ such that in the integrand the denominator elements of the infinite *q*-shifted factorials have modulus less than unity. Then7677

### Proof

First utilize the Ismail–Wilson generating function for Askey–Wilson polynomials (5) with

$$ (w,a,b,c,d,t)\mapsto ({\mathrm i},-{\mathrm i}a,-\tfrac{{\mathrm i}q}{a},{\mathrm i}b,\tfrac{{\mathrm i}q}{b},{\mathrm i}z). $$

An application of Andrews’ *q*-analogue of Whipple’s ${\phantom {a}}_3F_2(1)$ summation (16), followed by (c,e)↦(a,ab) and splitting the sum into the even and odd indexed parts completes the proof of (76). Finally, apply [11, Theorem 3.3] to (16) with $(a,b,c,d,t,z)\mapsto \big (-{\mathrm i}a,{\mathrm i}b,\tfrac{{\mathrm i}q}{b},-\tfrac{{\mathrm i}q}{a},{\mathrm i}z,{\mathrm i}\big )$, which produces (77), completing the proof. □

### Remark 4.11

The above transformation can also be obtained by starting with the Ismail–Wilson generating function (5) and applying Theorem 3.23.

A classic nonterminating *q*-product formula which generalizes T. Clausen’s (1828) formula [25, (16.12.2)], [9]was derived by F. H. Jackson (1940) [18], namelywhere |q|<1 and |z|<1. A *q*-analogue of a result given by Orr (1899) [5, (10.1.5)], [26]is derived in Nassrallah’s (1982) thesis, namely [24, (3.3.1)], given below in (78). For this product formula we provide an integral representation.

### Theorem 4.12

Let 0<|q|<1, $f,a,b,z\in {{\mathbb {C}}^*}$ with |z|<1, $w={\mathrm e}^{i\psi }$, $\sigma \in (0,\infty )$ such that in the integrand the denominator elements of the infinite *q*-shifted factorials have modulus less than unity. Then7879

### Proof

Start with [24, (3.3.1)] which gives (78). Then application of [11, Theorem 3.3] with

$$\begin{aligned} (a,b,c,d,t,z,q)\mapsto \big (q^\frac{1}{2}a^2,q^{-\frac{1}{2}}a^2,q^{\frac{1}{2}}b^2,q^{-\frac{1}{2}}b^2,q^{\frac{1}{2}}z, q^{\frac{1}{2}},q^2\big ) \end{aligned}$$

yields the integral representation (79), completing the proof. □

A *q*-analogue of another result given by Orr (1899) [5, (10.1.6)], [26]is derived in Nassrallah’s (1982) thesis, namely [24, (3.3.2)] (which we have corrected), that we give below in (80). We also provide an integral representation for this product formula.

### Theorem 4.13

Let 0<|q|<1, $f,a,b,z\in {{\mathbb {C}}^*}$ with |z|<1, $w={\mathrm e}^{i\psi }$, $\sigma \in (0,\infty )$ such that in the integrand the denominator elements of the infinite *q*-shifted factorials have modulus less than unity. Then8081

### Proof

Start with the corrected version of [24, (3.3.2)]. Then application of [11, Theorem 3.3] with

$$\begin{aligned} (a,b,c,d,t,z,q)\mapsto \big (q^{\frac{1}{2}}a^2,q^{-\frac{1}{2}}a^2,q^{\frac{1}{2}}b^2,q^{\frac{3}{2}}b^2,q^{\frac{1}{2}}z, q^{\frac{1}{2}},q^2\big ) \end{aligned}$$

yields the integral representation (81), completing the proof. □

### Remark 4.14

By starting with the the product generating function for Askey–Wilson polynomials (5) where $x=\frac{1}{2}(w+w^{-1})$, and replacing

$$\begin{aligned} (w,a,b,c,d)\mapsto \left( q^{-\frac{1}{4}}, q^{\frac{1}{4}}a,q^{-\frac{1}{4}}\tfrac{b}{a},q^{\frac{1}{4}}\tfrac{b}{a},q^{-\frac{1}{4}}a\right) , \end{aligned}$$

followed by using the basic hypergeometric representation for Askey–Wilson polynomials (2), the balanced ${\phantom {a}}_4\phi _3$ is converted into the form of the second *q*-analogue of Bailey’s ${\phantom {a}}_4F_3(1)$ sum, namely (19) [25, (17.7.13)], where $q^2$ is replaced with *q*. Then adopting the right-hand side of (19) and replacing $q\mapsto q^2$ in the entire expression and setting $t\mapsto q^\frac{1}{4}z$, followed by replacing $(a,b)\mapsto (qb^2,qa^2)$ produces (80).

### Remark 4.15

As pointed out to us by Slobodan Damjanovic, the *q*-quadratic summation in (78) is equivalent (by comparison of the coefficients of $z^n$ on both sides of the identity, etc.) to Guo’s summation in (20). Similarly, the *q*-quadratic summation in (80) is equivalent (by comparison of the coefficients of $z^n$ on both sides of the identity, etc.) to Wei and Wang’s summation in (23).

### Remark 4.16

If one sets $a^2=q$ in (80) or $b^2=q$ in (84) below, then one obtains(the series being convergent for |q|<1 and |z|<1). Note however that this result is trivially satisfied, as the identity holds termwise.

Another formula relating a product of two nonterminating ${\phantom {a}}_2\phi _1$ series with a nonterminating ${\phantom {a}}_4\phi _3$ series was obtained by Srivastava and Jain [32, (4.9)] (see also [29, Theorem 2.1]), given in (82) below. For this product formula we also provide an integral representation.

### Theorem 4.17

Let |q|<1, $f,a,b,z\in {{\mathbb {C}}^*}$ with |z|<1, $w={\mathrm e}^{i\psi }$, $\sigma \in (0,\infty )$ such that in the integrand, the denominator elements of the infinite *q*-shifted factorials have modulus less than unity. Then8283

### Proof

Start with (12). Application of [11, Theorem 3.3] with

$$\begin{aligned} (a,b,c,d,t,z)\mapsto (-{\mathrm i}a,{\mathrm i}b,-{\mathrm i}b,{\mathrm i}a,{\mathrm i}z,{\mathrm i}) \end{aligned}$$

yields (83), completing the proof. □

This formula is a *q*-analogue of Bailey’s formula [3, (2.11)]Along the lines of [29], we are able to derive similar product formulas. The first is given as follows.

### Theorem 4.18

Let 0<|q|<1, $f,a,b,z\in {{\mathbb {C}}^*}$ with |z|<1, $w={\mathrm e}^{i\psi }$, $\sigma \in (0,\infty )$ such that in the integrand the denominator elements of the infinite *q*-shifted factorials have modulus less than unity. Then8485

### Proof

First start with the Ismail–Wilson product generating function for Askey–Wilson polynomials (5), and apply

$$\begin{aligned} (w,a,b,c,d)\mapsto \big (q^{-\frac{1}{4}},q^{\frac{1}{4}}a,q^{-\frac{1}{4}}\tfrac{b}{a},q^{\frac{1}{4}}\tfrac{b}{a},q^{\frac{3}{4}}a\big ). \end{aligned}$$

Then using the basic hypergeometric representation for Askey–Wilson polynomials (2), the balanced ${\phantom {a}}_4\phi _3$ is converted into the form of the first *q*-analogue of Bailey’s ${\phantom {a}}_4F_3(1)$ sum, namely [25, (17.7.12)]86where $q^2$ is replaced with *q*. Then adopting the right-hand side of (86) and replacing $q\mapsto q^2$ in the entire expression and setting $t\mapsto q^\frac{1}{4}z$, followed by replacing $(a,b)\mapsto (qa^2,qb^2)$, product formula (85) is obtained. Replace $q^2\mapsto q$. Then the application of [11, Theorem 3.3] with

$$\begin{aligned} (a,b,c,d,t,z)\mapsto \big (q^\frac{1}{4}a^2,q^{-\frac{1}{4}}a^2,q^\frac{1}{4}b^2,q^{-\frac{1}{4}}b^2,q^\frac{1}{4}z,q^\frac{1}{4}\big ), \end{aligned}$$

followed by replacing $q\mapsto q^2$, yields the integral representation (85), completing the proof. □

### Corollary 4.19

Let $a,b,z\in {{\mathbb {C}}}$ such that |z|<1 and $2a+2b\not \in -\mathbb {N}$. Then87

### Proof

Start with Theorem 4.18 and replace $(a,b)\mapsto (q^a,q^b)$. Then take the limit as $(q^2,q)\rightarrow 1^{-}$ on the left and right-hand sides, respectively, followed by the replacement $(a,b)\mapsto (a+\frac{1}{2},b+\frac{1}{2})$, completing the proof. □

### Remark 4.20

If one starts with (5) and applies the substitution $(w,a,b,c,d)\mapsto ({\mathrm i},a,b,-a,-b)$ (notice that w=i or $w=-{\mathrm i}$ is equivalent to x=0), then the Askey–Wilson polynomial appearing in the series, written as a ${\phantom {a}}_4\phi _3$ using (4), can be summed using (15). Using the fact that only those terms of the series survive for which the summation index *n* is even (the other terms vanish), replacing *n* by 2*n*, followed by a series of simplifications, one arrives at the identitywhere |q|<1 and |t|<1 for convergence. This product formula was previously derived in Srivastava (1987) [31, (3.13)] (see also [29, (3.1)]).

By starting with Theorem 3.25 and inserting it into the Ismail–Wilson generating function (5) we obtain the following *q*-quadratic transformation for a product of two ${\phantom {a}}_2\phi _1$s given in terms of a sum of two *q*-quadratic ${\phantom {a}}_4\phi _3$’s.

### Theorem 4.21

Let 0<|q|<1, $a,b,z\in {{\mathbb {C}}^*}$ with |z|<1. Then88

### Proof

First start with the Ismail–Wilson product generating function for Askey–Wilson polynomials (5), and replace

$$\begin{aligned} (w,a,b,c,d)\mapsto ({\mathrm i}q^{\frac{1}{2}},\{\!\pm \!\}{\mathrm i}a,\{\!\pm \!\}{\mathrm i}b). \end{aligned}$$

Then using the Askey–Wilson summation formula Theorem 3.25, splitting the infinite sum into even and odd contributions and simplifying completes the proof. □

By starting with Theorem 3.26 and inserting it into the Ismail–Wilson generating function (5) we obtain the following *q*-quadratic transformation for a product of two ${\phantom {a}}_2\phi _1$s given in terms of a sum of two *q*-quadratic ${\phantom {a}}_5\phi _4$’s.

### Theorem 4.22

Let 0<|q|<1, $a,b,z\in {{\mathbb {C}}^*}$ with |z|<1. Then89

### Proof

First start with the Ismail–Wilson product generating function for Askey–Wilson polynomials (5), and replace

$$\begin{aligned} (w,a,b,c,d)\mapsto ({\mathrm i}q^{\frac{1}{2}},\{\!\pm \!\}{\mathrm i}a,{\mathrm i}b,-{\mathrm i}qb). \end{aligned}$$

Then using the Askey–Wilson summation formula Theorem 3.26, splitting the infinite sum into even and odd contributions and simplifying completes the proof. □

By starting with Theorem 3.27 and inserting it into the Ismail–Wilson generating function (5) we obtain the following transformation for a product of two ${\phantom {a}}_2\phi _1$’s given in terms of a sum of a ${\phantom {a}}_6\phi _5$’s and a ${\phantom {a}}_4\phi _3$ each with base $q^2$.

### Theorem 4.23

Let 0<|q|<1, $a,b,z\in {{\mathbb {C}}^*}$ with |z|<1. Then90

### Proof

First start with the Ismail–Wilson product generating function for Askey–Wilson polynomials (5), and replace

$$\begin{aligned} (w,a,b,c,d)\mapsto ({\mathrm i}q^{\frac{1}{2}},{\mathrm i}a,-{\mathrm i}qa,{\mathrm i}b,-{\mathrm i}qb). \end{aligned}$$

Then using the Askey–Wilson summation formula Theorem 3.27, splitting the infinite sum into even and odd contributions, scaling the variables appropriately and simplifying completes the proof. □

### Application of Cayley–Orr type expansion formulas

By using Cayley–Orr type expansion formulas stated in Gasper and Rahman’s textbook as [13, Exercises 3.17–3.19] (obtained by Singh [30] and Nassrallah [24]), one may verify the product formulas we have derived and derive new ones. We start with [13, Exercise 3.18] where we have replaced $(a,b)\mapsto (\frac{a}{q},\frac{b}{q})$.

#### Lemma 4.24

Let 0<|q|<1, $$a,b,c,z\in {{\mathbb {C}}^*}$$, |z|<1, $$|q^2cz|<|ab|$$ and91Then92

#### Theorem 4.25

Theorem 4.18 is verified by Lemma 4.24 with $c=\frac{ab}{q}$.

#### Proof

If one takes c=ab/q then the left-hand side of (92) becomes the left-hand side of (84). Replacing c=ab/q in (91) producesBy expanding the geometric series $(1-z)^{-1}$ then one can see that from (91), using the *q*-Pfaff–Saalschütz sum [13, (II.12)], that one obtains

$$\begin{aligned} a_n=\sum _{k=0}^n \frac{(\frac{a}{q},\frac{b}{q};q)_k}{(q,\frac{ab}{q};q)_k}q^k= \frac{(a,b;q)_n}{(q,\frac{ab}{q};q)_n}. \end{aligned}$$

Therefore from (92), we see thatwhich completes the verification using (1). □

Next we turn to [13, Exercise 3.19].

#### Lemma 4.26

Let 0<|q|<1, $$a,b,c,z\in {{\mathbb {C}}^*}$$, |z|<1, $$|q^2cz|<|ab|$$ and93Then94

#### Theorem 4.27

Nassrallah’s second result (80) is verified by Lemma 4.26 with c=qab.

#### Proof

If one takes $(a,b,c)\mapsto (qb,\frac{a}{q},qab)$ in Lemma 4.26, then the left-hand side of (94) becomes the left-hand side of (93). Replacing c=qab in (93) producesBy expanding the geometric series $(1-z)^{-1}$ then one can see that from (93), using the *q*-Pfaff–Saalschütz sum [13, (II.12)], that one obtains

$$\begin{aligned} a_n=\sum _{k=0}^n \frac{(\frac{a}{q},b;q)_k}{(q,ab;q)_k}q^k= \frac{(a,qb;q)_n}{(q,ab;q)_n}. \end{aligned}$$

Therefore from (94), we see thatwhich completes the verification using (1). □

Next we turn to [13, Exercise 3.17].

#### Lemma 4.28

Let 0<|q|<1, $a,b,c,z\in {{\mathbb {C}}^*}$, |z|<1, |cz|<|qab| and95Then96

#### Theorem 4.29

Let 0<|q|<1, $a,z\in {{\mathbb {C}}^*}$ such that |z|<1. Then one has the following three product formulas:979899

#### Proof

We apply Lemma 4.28 with suitable specializations of the parameters. Without yet specializing the parameters, we observe, that by virtue of the nonterminating *q*-binomial theorem, the coefficients $a_n$ in (95) can be computed as100the ${\phantom {a}}_4\phi _3$ series being terminating and 2-balanced. Comparing the above series with the terminating 2-balanced ${\phantom {a}}_4\phi _3$ summations derived in Sect. 3.1, we find that the coefficients $a_n$ reduce to closed form products by instances of Theorems 3.12, 3.13 and Corollary 3.16 in the three different cases $(a,b,c)\mapsto (a,-a,q^\sigma a^2)$, where $\sigma \in \{1,0,-1\}$. The computation in these three cases are as follows:

Case 1: $$(a,b,c)\mapsto (a,-a,q a^2)$$:

By Theorem 3.12 we have

$$\begin{aligned} a_n&=\frac{(-q;q^2)_n}{(q^2;q^2)_n}a^{2(n-2\lfloor \frac{n}{2}\rfloor )} \frac{(q;q^2)_{\lfloor \frac{n+1}{2}\rfloor }(-q^{1+2\lfloor \frac{n+1}{2}\rfloor }a^2;q^2)_{\lfloor \frac{n}{2}\rfloor }}{(qa^2;q^2)_{\lfloor \frac{n+1}{2}\rfloor }(-q^{1+2\lfloor \frac{n+1}{2}\rfloor };q^2)_{\lfloor \frac{n}{2}\rfloor }}\\&=a^{2(n-2\lfloor \frac{n}{2}\rfloor )} \frac{(q;q^2)_{\lfloor \frac{n+1}{2}\rfloor }(-q;q^2)_{\lfloor \frac{n+1}{2}\rfloor }(-q^{1+2\lfloor \frac{n+1}{2}\rfloor }a^2;q^2)_{\lfloor \frac{n}{2}\rfloor }}{(q^2;q^2)_n(qa^2;q^2)_{\lfloor \frac{n+1}{2}\rfloor }}\\&=a^{2(n-2\lfloor \frac{n}{2}\rfloor )} \frac{(q^2;q^4)_{\lfloor \frac{n+1}{2}\rfloor }(-q^{1+2\lfloor \frac{n+1}{2}\rfloor }a^2;q^2)_{\lfloor \frac{n}{2}\rfloor }}{(q^2;q^2)_n(qa^2;q^2)_{\lfloor \frac{n+1}{2}\rfloor }}\\&=a^{2(n-2\lfloor \frac{n}{2}\rfloor )} \frac{(-q^{1+2\lfloor \frac{n+1}{2}\rfloor }a^2;q^2)_{\lfloor \frac{n}{2}\rfloor }}{(q^4;q^4)_{\lfloor \frac{n}{2}\rfloor }(qa^2;q^2)_{\lfloor \frac{n+1}{2}\rfloor }}\\&=a^{2(n-2\lfloor \frac{n}{2}\rfloor )} \frac{(-qa^2;q^2)_n}{(q^4;q^4)_{\lfloor \frac{n}{2}\rfloor }(qa^2;q^2)_{\lfloor \frac{n+1}{2}\rfloor }(-qa^2;q^2)_{\lfloor \frac{n+1}{2}\rfloor }}\\&=a^{2(n-2\lfloor \frac{n}{2}\rfloor )} \frac{(-qa^2;q^2)_n}{(q^4;q^4)_{\lfloor \frac{n}{2}\rfloor }(q^2a^4;q^4)_{\lfloor \frac{n+1}{2}\rfloor }}. \end{aligned}$$

Case 2: $$(a,b,c)\mapsto (a,-a,a^2)$$:

By Theorem 3.13 we have

$$\begin{aligned} a_n&=\frac{(-1;q^2)_n}{(q^2;q^2)_n}(-1)^n\frac{(q^n+q^{-n})}{2} \frac{(q;q^2)_{\lfloor \frac{n+1}{2}\rfloor }(-q^{2\lfloor \frac{n+1}{2}\rfloor }a^2;q^2)_{\lfloor \frac{n}{2}\rfloor }}{(qa^2;q^2)_{\lfloor \frac{n}{2}\rfloor }(-q^{2+2\lfloor \frac{n}{2}\rfloor };q^2)_{\lfloor \frac{n+1}{2}\rfloor }}\\&=(-1)^n q^{-n} \frac{(-q^2;q^2)_n(q;q^2)_{\lfloor \frac{n+1}{2}\rfloor }(-q^{2\lfloor \frac{n+1}{2}\rfloor }a^2;q^2)_{\lfloor \frac{n}{2}\rfloor }}{(q^2;q^2)_n(qa^2;q^2)_{\lfloor \frac{n}{2}\rfloor }(-q^{2+2\lfloor \frac{n}{2}\rfloor };q^2)_{\lfloor \frac{n+1}{2}\rfloor }}\\&=(-1)^n q^{-n} \frac{(-q^2;q^2)_{\lfloor \frac{n}{2}\rfloor }(q;q^2)_{\lfloor \frac{n+1}{2}\rfloor }(-a^2;q^2)_{n}}{(q^2;q^2)_n(qa^2;q^2)_{\lfloor \frac{n}{2}\rfloor }(-a^2;q^2)_{\lfloor \frac{n+1}{2}\rfloor }}\\&=(-q)^{-n} \frac{(q;-q)_n(-a^2;q^2)_{n}}{(q^2;q^2)_n(-a^2;-q)_n}, \end{aligned}$$

which remarkably involves base -q (“minus *q*", which is not a mistake!) instead of the usual base *q*.

Case 3: $$(a,b,c)\mapsto (a,-a,q^{-1} a^2)$$:

By Corollary 3.16 we have

$$\begin{aligned} a_n&=\frac{(-q^{-1};q^2)_n}{(q^2;q^2)_n} (-1)^n\frac{(1+q^{1-2n})}{(1+q)} \frac{(q;q^2)_{\lfloor \frac{n+1}{2}\rfloor }(-q^{-1+2\lfloor \frac{n+1}{2}\rfloor }a^2;q^2)_{\lfloor \frac{n}{2}\rfloor }}{(q^{-1}a^2;q^2)_{\lfloor \frac{n+1}{2}\rfloor }(-q^{1+2\lfloor \frac{n+1}{2}\rfloor };q^2)_{\lfloor \frac{n}{2}\rfloor }}\\&=(-1)^n q^{-2n} \frac{(-q;q^2)_n(q;q^2)_{\lfloor \frac{n+1}{2}\rfloor }(-q^{-1+2\lfloor \frac{n+1}{2}\rfloor }a^2;q^2)_{\lfloor \frac{n}{2}\rfloor }}{(q^2;q^2)_n(q^{-1}a^2;q^2)_{\lfloor \frac{n+1}{2}\rfloor }(-q^{1+2\lfloor \frac{n+1}{2}\rfloor };q^2)_{\lfloor \frac{n}{2}\rfloor }}\\&=(-1)^n q^{-2n} \frac{(-q;q^2)_{\lfloor \frac{n+1}{2}\rfloor }(q;q^2)_{\lfloor \frac{n+1}{2}\rfloor }(-q^{-1}a^2;q^2)_{n}}{(q^2;q^2)_n(q^{-1}a^2;q^2)_{\lfloor \frac{n+1}{2}\rfloor }(-q^{-1}a^2;q^2)_{\lfloor \frac{n+1}{2}\rfloor }}\\&=(-1)^n q^{-2n} \frac{(q^2;q^4)_{\lfloor \frac{n+1}{2}\rfloor }(-q^{-1}a^2;q^2)_{n}}{(q^2;q^2)_n(q^{-2}a^4;q^4)_{\lfloor \frac{n+1}{2}\rfloor }}\\&=(-1)^n q^{-2n} \frac{(-q^{-1}a^2;q^2)_{n}}{(q^4;q^4)_{\lfloor \frac{n}{2}\rfloor }(q^{-2}a^4;q^4)_{\lfloor \frac{n+1}{2}\rfloor }} . \end{aligned}$$

With these formulas for $a_n$ we can write out (96) in the respective cases. For the first case, after replacing *q* by $q^{\frac{1}{2}}$ amd *a* by $\sqrt{a}$, this yields (97). In the second case, after replacing *z* by -qz, and subsequently *q* by $q^{\frac{1}{2}}$ and *a* by $\sqrt{a}$, this yields (98). Finally, In the third case, after replacing *z* by $-q^2z$, and subsequently *q* by $q^{\frac{1}{2}}$ and *a* by $\sqrt{a}$, this yields (99). □

We offer another application of Lemma 4.28, similar to those applications in Theorem 4.29 but depending on a additional parameter and where the right-hand side involves a sum of two series.

#### Theorem 4.30

Let 0<|q|<1, $a,b,z\in {{\mathbb {C}}^*}$ such that |z|<1. Then one has the following product formula:101

#### Proof

The analysis is similar to that in the proof of Theorem 4.29. Again, we apply Lemma 4.28 but now with the specialization $c=q^2ab$. The coefficients in (95) can be computed by the $c=q^2ab$ case of (100), thuswhich is specific terminating 2-balanced ${\phantom {a}}_3\phi _2$ series. The latter can be evaluated by the $c=q^2ab$ case of the general terminating 2-balanced ${\phantom {a}}_3\phi _2$ summation, i.e.,which itself readily follows from the contiguous relation [23, (2.3)], in combination with the *q*-Pfaff–Saalschütz summation [13, (II.12)]. We thus obtain

$$\begin{aligned} a_n= \frac{(1-q)(1-qab)}{(1-qa)(1-qb)} \frac{(qa,qb;q)_{n}}{(q,qab;q)_{n}} +\frac{q\,(1-a)(1-b)}{(1-qa)(1-qb)} \frac{(qa,qb;q)_{n}}{(q,q^2ab;q)_{n}}. \end{aligned}$$

Writing out (96) for $c=q^2ab$ and this choice of $a_n$ gives (101). □

One can also derive other product formulas using the method employed in this section. One interesting example is obtained by the following slight modification of the analysis that was used to derive Theorem 4.27.

#### Remark 4.31

If one follows the same procedure as in the proof of Theorem 4.27, but instead takes $(a,b,c)\mapsto (\frac{a}{q},qb,qab)$ in Lemma 4.26, one obtains a different $a_n$, namely

$$\begin{aligned} a_n=\frac{(q^2b,\frac{a}{q};q)_n}{(q,ab;q)_n}. \end{aligned}$$

This is obtained using a different instance of the *q*-Pfaff–Saalschütz summation [13, (II.12)]. Following the same procedure as in the proof of Theorem 4.27 produceswhere |q|<1 and |z|<1 for convergence. However, this result can be seen to be equivalent to Nassrallah’s second result (80) by suitably shifting *a* and *b*.

## Conclusion

In this paper we focused on known and new results related to the Ismail–Wilson generating function for Askey–Wilson polynomials (5). In Theorem 2.3 we were able to give an extension of (5) that contains an extra parameter *u* (and reduces to the former if u=0), which we used to deduce a quadruple basic hypergeometric summation formula in Corollary 2.4. Independently, by specializing the Ismail–Wilson generating function (5) in different ways, such that the Askey–Wilson polynomials that appear in the expansion simplify (by virtue of existing summations for terminating balanced ${\phantom {a}}_4\phi _3$ series), we were able to identify a number of formulas for products of ${\phantom {a}}_2\phi _1$ basic hypergeometric series (some of them known, some of them new) – for which we also provided corresponding integral representations. In our effort to put the results obtained into context, we also described some alternative ways to arrive at several of the results, such as invoking Cayley–Orr type expansion formulas. By applying meaningful variations of those derivations, we succeeded in finding yet further new identities. Altogether, we believe that the material provided in this paper forms a valuable contribution to identities satisfied by (specialized) Askey–Wilson polynomials.

## Data Availability

No datasets were generated or analysed during the current study.
